# Health risk assessment of enrofloxacin, oxytetracycline, and sulfadimidine residues in broiler chicken carcasses marketed in Mit Ghamr, Egypt

**DOI:** 10.1038/s41598-026-63227-2

**Published:** 2026-07-29

**Authors:** Sherine Mohsen Noah, Samir Mohammed Abd-Elghany, Khalid Ibrahim Sallam

**Affiliations:** Department of Food Hygiene, Safety, and Technology, Faculty of Veterinary Medicine, Mansoura University, Mansoura, 35516 Egypt

**Keywords:** HPLC, Antimicrobial residues, Chicken meat, Chicken liver, Health hazard, Diseases, Health care, Medical research, Microbiology, Zoology

## Abstract

The widespread use of antibiotics in poultry production raises concerns about drug residues in chicken tissues and associated public health risks. Chicken tissue samples (*n* = 180; 60 each from breast, thigh, and liver) were purchased from various poultry shops in Egypt and analyzed using high-performance liquid chromatography (HPLC). Residues of enrofloxacin, oxytetracycline, and sulfadimidine were detected in 51.67% (93/180), 71.67% (129/180), and 16.67% (30/180) of chicken samples, respectively. Interestingly, 54.84% (51/93) of enrofloxacin-positive samples, 55.81% (72/129) of oxytetracycline-positive samples, and 70% (21/30) of sulfadimidine-positive samples exceeded the maximum residue limits (MRLs). Among the 180 samples of each of chicken breast, thigh, and liver samples, 5% of each tissue type contained the three antibiotics tested, while 40% of chicken breasts and 50% of both thighs and liver samples contained two of the three antibiotics. HPLC analysis revealed mean concentrations of 90.4, 317.7, and 573.3 µg/kg for enrofloxacin, oxytetracycline, and sulfadimidine, respectively, in chicken breasts; 161.5, 964.1, and 47, respectively, in chicken thighs; and 1707.3, 10,625, and 3848.1, respectively, in livers. The estimated daily intake (EDI) for antibiotics analyzed in chicken tissue among consumers was between 0.012 and 0.590 µg/kg body weight/day. The Hazard quotient (HQ) values of enrofloxacin, oxytetracycline, and sulfadimidine ranged between 0.0006 and 0.050 for schoolchildren, 0.0003 and 0.022 for women, and 0.0002 and 0.021 for men, respectively. Among the different age groups of the Egyptian consumers, schoolchildren had the highest daily intake and hazard quotient values of antibiotic residues from chicken tissue. The HQ values for tested antibiotics indicated no significant health risk for either adults or children, although children showed relatively higher values. Overall, the findings highlight the need for strict antibiotic monitoring, with consideration of their withdrawal periods in the veterinary field, to ensure food safety and to protect public health.

## Introduction

Chicken meat is widely accepted as a healthy, lean, protein-rich, nutrient-dense, versatile, and low-fat (2.5–10%) food, composed mainly of unsaturated fatty acids. It provides vitamins (B vitamins) and minerals (iron, zinc, phosphorus, potassium) and is generally lower in fat and cholesterol than red meat, making it a popular choice for balanced diets and muscle building. It also faces a few cultural or religious restrictions^1,2^. Despite its health benefits, chicken meat may pose potential risks due to the frequent use of antimicrobial agents (mainly antibiotics) as feed supplements in birds to promote growth, increase weight and productivity, and treat or prevent diseases. Inappropriate use can lead to these antibiotics accumulating as residues in the tissues, caused by illegal overdosing, insufficient withdrawal periods, or a lack of understanding of drug use^3–5^. Such antimicrobial residues might exceed the maximum permissible residue limits and contribute to the development of antimicrobial-resistant microorganisms, which may subsequently cause human infections that are difficult or sometimes impossible to treat.

Currently, the estimated global average annual consumption of antimicrobials for chicken is 35.4 mg/kg^6^. Antibiotics such as enrofloxacin, oxytetracycline, and sulfadimidine are frequently used in developing countries in poultry feed. Enrofloxacin is a second-generation bactericidal fluoroquinolone that exhibits broad-spectrum activity against both Gram-positive and Gram-negative bacteria and is widely used in animals and poultry to treat respiratory, urinary, gastrointestinal, and skin infections^7–9^. Oxytetracycline is one of the broad-spectrum bacteriostatic antibiotics derived from tetracyclines. It is commonly used in veterinary medicine due to its low cost and ease of administration via drinking water or feed^10^. Unfortunately, inadequate withdrawal periods and the abuse of oxytetracycline antibiotics in chicken production have led to residues that may pose risks to human health^11^, including teratogenic effects in the fetus and hypoplasia of developing teeth when infants are exposed^12^. Sulfonamides, on the other hand, are widely used in veterinary medicine as prophylaxis therapy for many diseases in chickens such as infectious coryza, coccidiosis, fowl typhoid, and pullorum disease^13,14^. Sulfadimidine is commonly used as a feed additive in chicken production to control infections and promote growth due to its low cost and high efficacy^15^.

Enrofloxacin, oxytetracycline, and sulfadimidine were selected in the present study because they represent three major classes of antimicrobials widely used in poultry production, namely fluoroquinolones, tetracyclines, and sulfonamides. These antibiotics are commonly administered for disease prevention and treatment in broiler chickens and are frequently reported as residues in poultry products. Therefore, they serve as important indicators for monitoring antimicrobial residue contamination and assessing potential public health risks associated with poultry consumption.

Antimicrobial residues in foods of animal origin, including meat, milk, eggs, and other products, can pose serious health risks to humans when they exceed maximum allowable residual limits. Even at comparatively low doses, these substances may cause direct toxic effects, promote the emergence of resistant microorganisms, and trigger allergic reactions^16^. These health risks include immunopathological reactions, carcinogenic effects (associated with sulfamethazine, oxytetracycline, and furazolidone), mutagenic potential, kidney damage such as nephropathy (notably with gentamicin), liver toxicity, reproductive impairments, bone marrow suppression (linked to chloramphenicol), and severe allergic reactions, including anaphylactic shock^17^.

Many researchers have documented multiple adverse health effects linked to the presence of drug residues in foods, including poultry products^18–21^. The most significant adverse consequence of antibiotic residues in food is the emergence of antibiotic-resistant bacteria, which reduces the effectiveness of antibiotics against infections that were previously treatable^22^. Current estimates indicate that antibiotic-resistant bacteria cause approximately 700,000 deaths worldwide each year, in addition to prolonged illness with longer hospital stays, disability, higher medication costs, and increased mortalities. The predictions suggest mortalities will rise to 10 million annually by 2050^23,24^.

The widespread use of antimicrobial agents has become a significant threat to public health and food safety. The presence of antimicrobial residues in food products contributes to the development of antimicrobial resistance, posing risks to consumers worldwide. To address these concerns, international health organizations such as the World Health Organization (WHO) and the Food and Agriculture Organization (FAO) have established guidelines for acceptable daily intake (ADI) and maximum residue limits (MRLs) to ensure consumer safety. Moreover, the Codex Alimentarius Commission created the Codex Committee on Residues of Veterinary Medications in Food, which set MRLs for about 72 veterinary drugs^25^. Several countries, including the United States of America (USA), Canada, the European Union (EU), etc., have also established their own maximum residue limits^26^.

Therefore, monitoring is necessary to confirm that antibacterial substances are not present at levels that could threaten public health or compromise food safety. A wide range of analytical approaches are available for detecting antibiotic residues in poultry tissues, including chromatographic methods such as thin-layer chromatography (TLC), high-performance liquid chromatography (HPLC), and capillary electrophoresis (CE). These techniques are highly sensitive, accurate, and quantitative, providing a reliable determination of antimicrobial residues in animal muscles and organs^26^. This study was therefore designed to quantitatively evaluate the presence of antibiotic residues of three widely used antibiotics (enrofloxacin, oxytetracycline, and sulfadimidine) in edible tissues and organs (breast, thigh, and liver) of broiler chicken carcasses commercially marketed in Egypt, along with the assessment of the potential risks associated with consuming meat containing these antibiotic residues.

## Materials and methods

### Chicken sampling and study design

A total of 180 chicken tissue samples, consisting of 60 breast, 60 thigh, and 60 liver samples, were randomly collected from different poultry shops in Mit Ghamr City, Egypt, between September and March 2025. The average weight of each dressed carcass was about 1.25 kg. From breast and thigh tissues, about 50 g was sampled and stored at − 20 °C until preparation for analysis, while the whole liver was sampled. High-performance liquid chromatography (HPLC) was employed in this study to determine the antibiotic residue levels in the collected tissues.

### Chemicals and materials

HPLC-grade standards of enrofloxacin (ENR), oxytetracycline (OTC), and sulfadimidine (SDI) were obtained from Sigma-Aldrich (Merck KGaA, Darmstadt, Germany). Analytical grade citric acid monohydrate, formic acid, nitric acid, and potassium dihydrogen phosphate were obtained from Himedia (India). HPLC-grade water, methanol (MeOH), and acetonitrile (ACN) were purchased from POCH SA (Gliwice, Poland).

### Standard stock solutions preparation

Aqueous stock solutions of 100 µg/mL were prepared by taking 5 mg of each ENR, OTC, and SDI standard in a 50-mL volumetric flask and making up to the mark with acetonitrile and phosphoric acid buffer (for ENR) or pure methanol (for OTC and SDI). Stock solutions were stored at 4 °C for up to 4 weeks in an amber-colored glass bottle. Working solutions were prepared by appropriate dilution of aliquots of the standard stock solutions in HPLC-grade organic solvents. The working solutions were used to prepare calibration curves at concentrations of 0.05, 0.1, 0.2, 0.5, 1, 2, 5, 10, 20, and 50 µg/mL for different antibiotics, using the same dilution solvents.

### Sample extraction

Extraction and clean-up procedures were performed for all frozen chicken samples to be further examined by HPLC for quantitative determination of residues of the three selective antimicrobials. For the determination of enrofloxacin, oxytetracycline, and sulfadimidine residues, frozen tissue samples were first thawed, trimmed of external fat and fascia, finely chopped, and homogenized after weighing 2 g of each tissue.

For enrofloxacin analysis, the homogenized sample was mixed with 8 mL of 5% trichloroacetic acid, vortexed for 1 min, shaken for 10 min, centrifuged at 14,000 rpm for 5 min at 4 °C, filtered through a 0.45 μm nylon filter, and 20 µL of the filtrate was injected into the HPLC system^27^.

For oxytetracycline determination, the homogenized tissue was blended for 2 min, followed by addition of 0.1 g citric acid, 1 mL of 30% nitric acid, 4 mL methanol, and 1 mL deionized water; the mixture was vortexed, sonicated for 15 min, centrifuged at 5300 rpm for 10 min, filtered through a 0.45 μm nylon filter, and 20 µL was injected into HPLC^12^.

For sulfadimidine analysis, the homogenized sample was transferred to a 15 mL polypropylene tube, extracted with 5 mL acetonitrile containing 0.2% formic acid, vortexed for 1 min, sonicated for 30 min, and centrifuged at 8000 rpm for 5 min. After degreasing, the supernatant was filtered and subjected to solid-phase extraction (SPE) using cartridges preconditioned with methanol and water. Then 20 µL of the eluent was injected into the HPLC for analysis^28^.

### Quantitative analysis by High-performance liquid chromatography (HPLC)

#### Analysis of antibiotic residues using the HPLC technique

For the chromatographic determination of ENR, OTC, and SDI, the Surveyor HPLC system was controlled by Chromo Quest version 4.2 software for data analysis (Thermo Fisher Scientific Inc., Waltham, MA, USA). A consistent liquid chromatography pump (Surveyor, Thermo Scientific, USA) was used to enable automated operation, delivering the solvent system to the analytical column. An Eclipse 5 μm XDB C18, 150 × 4.6 mm analytical column (Agilent Inc., Santa Clara, CA, USA) was used. The detection was performed using a PDA detector (Surveyor, Thermo Scientific, USA) at a wavelength of 278 nm for ENR, 350 nm for OTC, and 264 nm for SDI. Mobile phases were degassed using syringe filters with 0.45 mm MCE membranes (Carrigtwohill company, Cork, Ireland) to ensure sample purity, and an ultrasonic bath (3510, Branson, USA) was used to facilitate solute dissolution. The chromatographic conditions were determined for HPLC analysis of ENR, OTC, and SDI residues (Table 1).

**Table 1:** Chromatographic conditions for HPLC for the determination of enrofloxacin (ENR), oxytetracycline (OTC), and sulfadimidine (SDI).

HPLC system	Chromatographic conditions		
	Enrofloxacin	Oxytetracycline	Sulfadimidine
Column	XDB C18 (5 μm, 150 × 4.6 mm)	XDB C18 (5 μm, 150 × 4.6 mm)	XDB C18 (5 μm, 150 × 4.6 mm)
Solvent system	Acetonitrile and phosphoric acid buffer (0.01 M, pH 3)	Methanol, formic acid (0.1%), and Acetonitrile	Potassium di-hydrogen phosphate buffer (0.01 M) and methanol
Solvent system ratio	25:75% v/v	20:70:10 v/v/v	70:30 v/v
Injection volume (µL)	20 µL	20 µL	20 µL
Flow rate (mL/min)	1	1	1
Pump pressure (kg/m^2^)	134–137	128–135	130–139
Wavelength	278 nm	350 nm	264 nm
Retention time (T_R_) (min)	6.2 ± 0.04	5.4 ± 0.03	6.5 ± 0.04
Run time (min)	10	10	10
Temperature	25 °C	25 °C	25 °C

####  HPLC method validation

The HPLC-PDA analytical method was validated by evaluating key performance characteristics, including linearity, limits of detection (LOD) and quantification (LOQ), accuracy, precision, and selectivity. These parameters were determined individually for each target antibiotic in chicken meat samples in accordance with the standards established by European Commission Decision 657/2002/EC^29^.

Linearity was evaluated using 5 solvent-matched calibration levels analyzed in triplicate for each analyte. The concentration ranges inspected were 0.1–20 µg mL^−1^ for enrofloxacin, 0.1–50 µg mL^−1^ for oxytetracycline, and 0.05–20 µg mL^−1^ for sulfadimidine. Calibration curves were constructed by linear regression of peak area versus analyte concentration, and linearity was expressed as the coefficient of determination (R^2^). Method sensitivity was estimated according to the International Conference on Harmonization (ICH) guidelines by calculating the limits of detection (LOD) and quantification (LOQ) using the following equations: LOD = 3.3σ/m and LOQ = 10σ/m, where σ is the residual standard deviation of the regression and m is the slope of the calibration curve^30^.

### Characterization of health risks associated with exposure to chicken samples containing antibiotic residues

#### Determination of estimated daily intake (EDI)

The estimated daily intake (EDI) of antibiotic residues from chicken meat consumption was calculated based on antibiotic concentrations in chicken meat samples, daily dietary patterns, and body weight. EDI was assessed for different age groups using the following equation: EDI = MC × DC/BW, where MC represents the mean concentration of antibiotic residues in chicken meat (µg/kg), DC is the daily consumption of chicken meat, and BW is body weight (kg).

The average body weight in different age groups of the Egyptian population was estimated to be 34 kg for schoolchildren at the ages between 5 and 14 years based on Egyptian growth charts (https://ia804602.us.archive.org/2/items/egyptian-growth-charts/Egyptian%20growth%20charts.pdf), and 77.1 kg for women and 79.1 kg for men at the ages between 15 and 59 years as reported by the Ministry of Health and Population, Cairo, Egypt (https://dhsprogram.com/pubs/pdf/FR313/FR313.pdf).

#### Determination of hazard quotient (HQ)

The estimated daily intake (EDI) was compared with the acceptable daily intake (ADI) set by JECFA^31^ to calculate the hazard quotient (HQ) for the detected antibiotics using the following equation: HQ = EDI/ADI. The average daily intake (ADI) for ENR is 2 µg/kg.bw /day; for OTC is 30 µg/kg.bw/day; and for SDI is 50 µg/kg. bw/day. The HQ acts as a screening indicator for risk assessment and reflects the potential health risk associated with exposure to the compounds under investigation.

### Statistical analysis

Antimicrobial residue measurements in the chicken samples were determined in triplicate. Statistical analysis was carried out using the Vassar Stats online tool (http://vassarstats.net). Differences in antimicrobial levels among the various chicken tissues were assessed using one-way analysis of variance (ANOVA), followed by Tukey’s Honestly Significant Difference (HSD) test, with significance considered at *P* < 0.05 or *P* < 0.01.

**Fig. 1:**
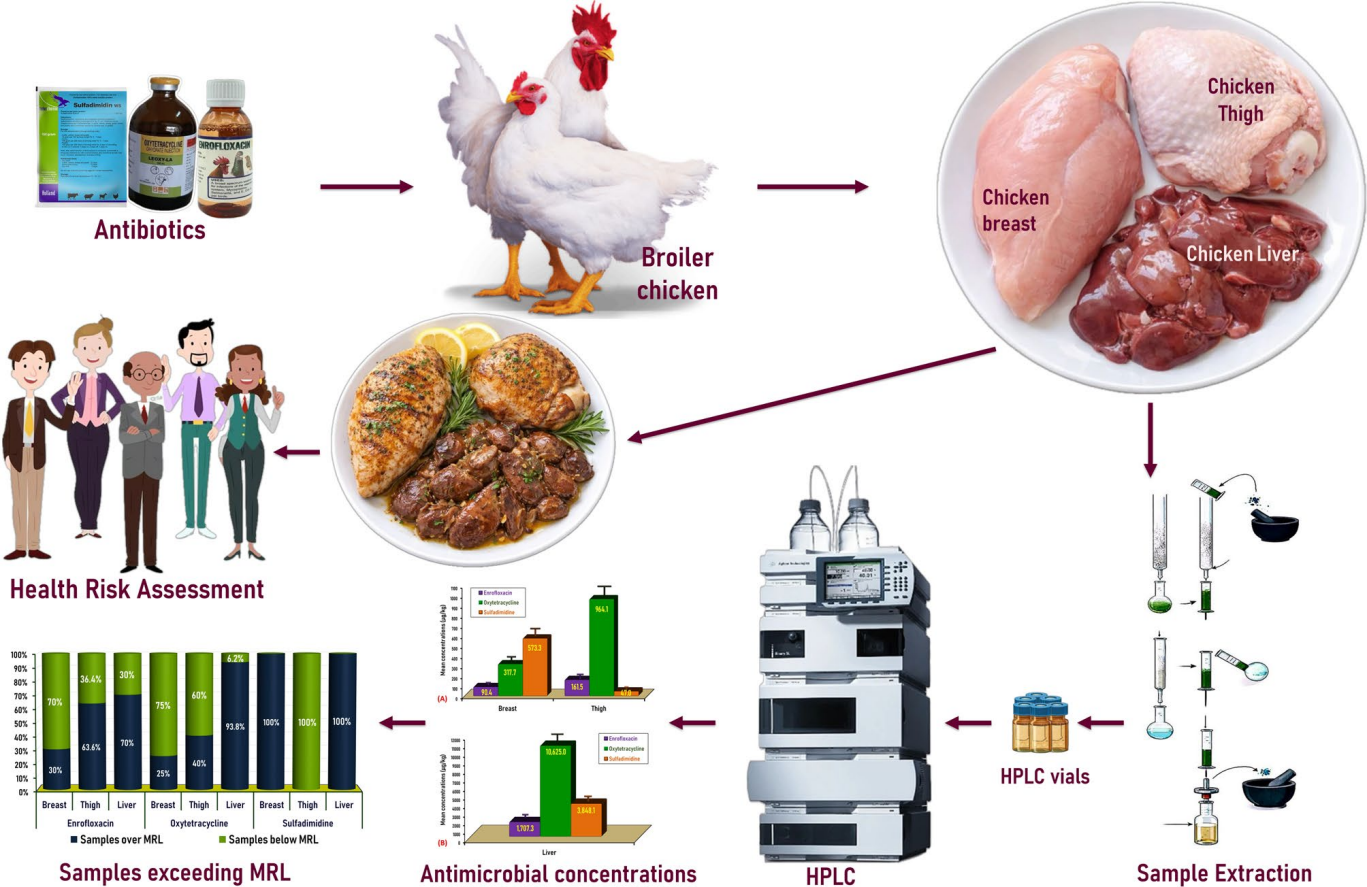
A graphical abstract illustrates the experimental design showing the antibiotics used in broiler chickens, HPLC-based determination of enrofloxacin, oxytetracycline, and sulfadimidine residues in thigh, breast, and liver tissues, evaluating the maximal residue limits (MRLs) exceedances, and subsequent human health risk assessment.

A graphical abstract summarizing the study presented in this work is shown in Fig. 1.

## Results and discussion

### Validation and method performance of HPLC

**Fig. 2:**
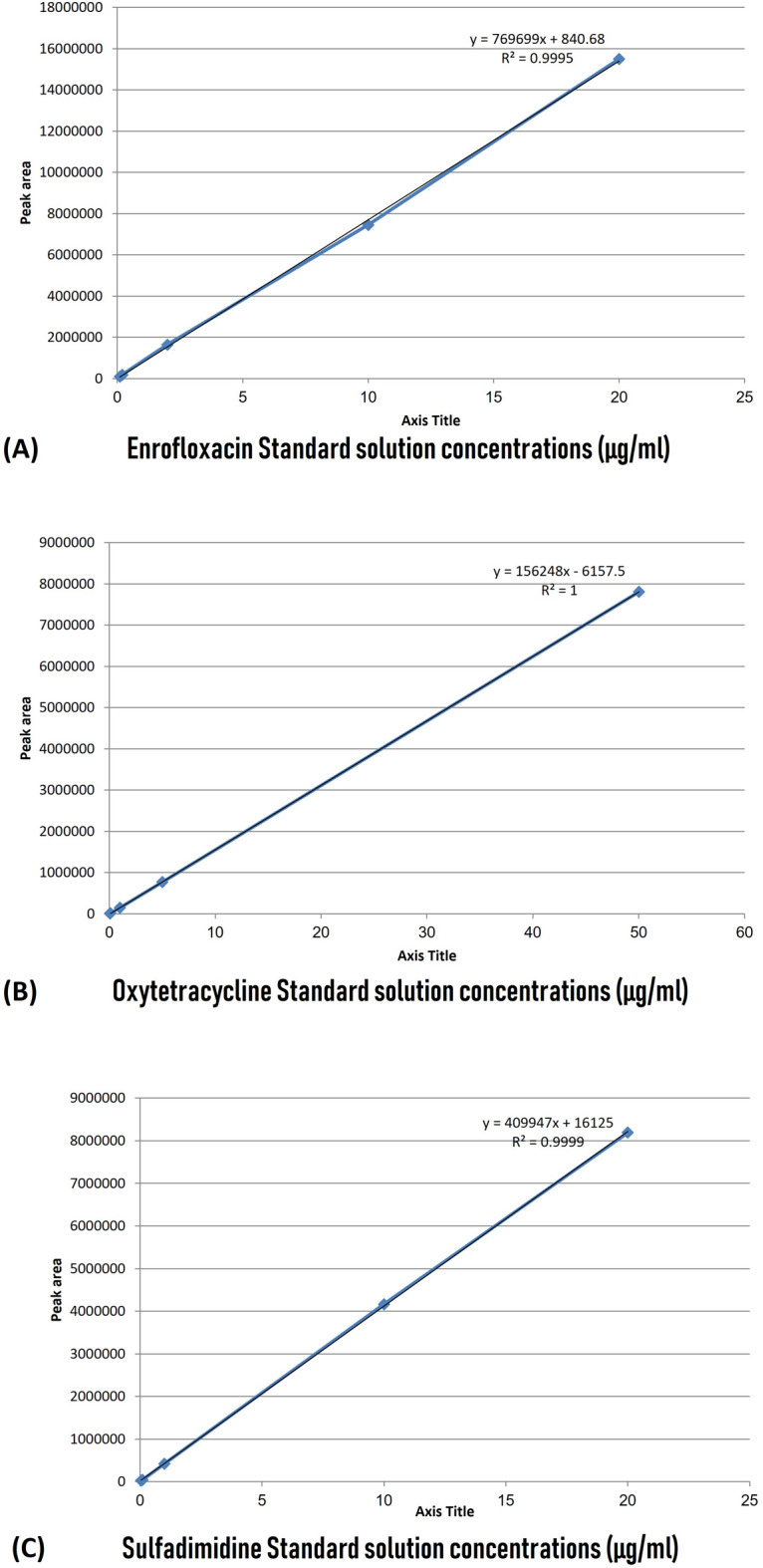
HPLC standard calibration curves for (**A**): Enrofloxacin (0.1, 0.2, 2, 10, and 20 µg/mL), (**B**): Oxytetracycline (0.1, 0.5, 1, 5, and 50 µg/mL), and (**C**): Sulfadimidine (0.05, 0.1, 1, 10, and 20 µg/mL).

**Fig. 3:**
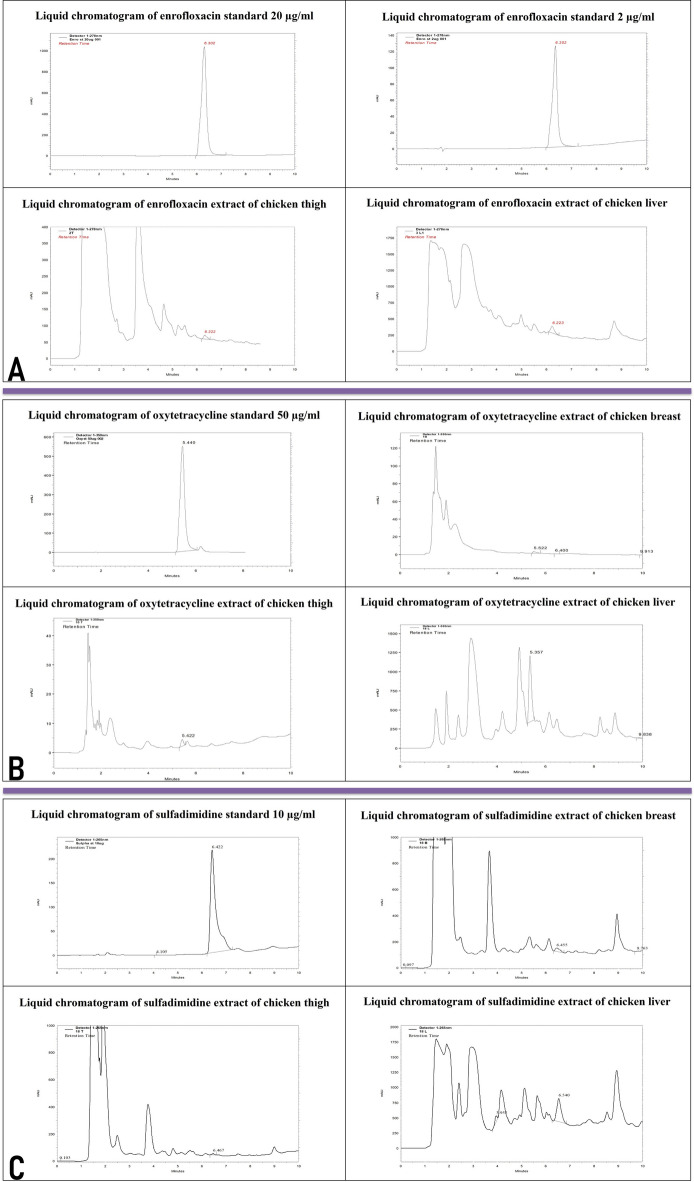
Liquid chromatograms of the standards and chicken sample extracts for tested antibiotics. Liquid chromatograms of enrofloxacin standard and chicken sample extract (**A**). Liquid chromatograms of oxytetracycline standard and chicken sample extract (**B**). Liquid chromatograms of sulfadimidine standard and chicken sample extract (**C**).

The linearity, limits of detection (LOD), limits of quantification (LOQ), recovery, intraday, and inter-day precisions of the HPLC-PDA method for the analyzed antibiotics are shown in Table 2. Sample preparation procedures and mobile phase compositions reported in previous studies for determining antibiotic residues in chicken samples were adopted to optimize the HPLC-PDA conditions in the present investigation^32–34^. High peak resolution and reliable identification of the investigated antibiotics were achieved by applying isocratic and gradient elution conditions at a mobile phase flow rate of 1 mL min^−1^. All method validation parameters met the European Commission regulatory requirements^29^. Five-point solvent-matched calibration curves exhibited linearity over the concentration range of 0.1–20 µg mL^−1^ for enrofloxacin, 0.1–50 µg mL^−1^ for oxytetracycline, and 0.05–20 µg mL^−1^ for sulfadimidine, with coefficients of determination (R^2^) of 0.9995, 1, and 0.9999 for enrofloxacin, oxytetracycline, and sulfadimidine, respectively (Fig. 2). The recovery values were 93–97%, 96–99%, and 92–96% for ENR, OTC, and SDI, respectively with the intraday precision varied between 1.6% and 4.3% and inter day precision between 0.8 and 1.9 for tested analytes (Table 2). Meanwhile, the recovery values and RSD% complied with the European Commission limits of 70–120% and < 20%, respectively^29^. The optimized and validated HPLC-PDA method applied in this study was deemed accurate and precise. In addition, the HPLC-PDA chromatograms exhibited good resolution, with no interfering matrix peaks observed within the retention time screens of the tested analytes (Fig. 3). Moreover, the retention times were 6.2, 5.4, and 6.5 min for ENR, OTC, and SDI, respectively, indicating the selectivity of the detection and confirmation methods of antibiotics in chicken meat samples.

**Table 2:** Validation sheet of the HPLC analytical method.

Parameter	Enrofloxacin	Oxytetracycline	Sulfadimidine
Calibration curve range (µg/mL)	0.1, 0.2, 2, 10, 20	0.1, 0.5, 1, 5, 50	0.05, 0.1, 1, 10, 20
Retention time (min)	6.2 ± 0.04	5.4 ± 0.03	6.5 ± 0.04
Linear regression (equation)	y = 769699x + 840.68	y = 156248x − 6157.5	y = 409947x + 16,125
Linearity (R^2^)	0.9995	1	0.9999
Slope (a)	769,699	156,248	409,947
Intercept (b)	840.68	6157.5	16,125
Limit of detection LOD (µg/kg)	5.45	9	1.6
Limit of quantification (µg/kg)	18.8	30	5.05
Recovery%	93–97%	96–99%	92–96%
Intraday precision (RSD%)	1.6	2.3	4.3
Inter-day precision (RSD%)	0.8	1.2	1.9

A limitation of the present study is that antimicrobial residues were quantified using HPLC without confirmatory LC-MS/MS analysis. Although the validated HPLC method provided reliable quantitative results, future investigations employing LC-MS/MS are recommended to further confirm the identity and concentrations of the detected residues.

### Prevalence and concentration of enrofloxacin residues (µg/kg) in chicken breast, thigh, and liver samples

HPLC analysis of poultry tissue samples measured the presence of antimicrobial residues and concentrations (µg/kg) of the detected antimicrobials (Table 3). The analysis also presented the maximum residue limits (MRLs) according to the Codex Alimentarius^25^, as well as the number of samples that were below or exceeded these limits. Furthermore, a comparative representation of the relative proportions of positive samples exceeding the MRLs and those below the permissible limits are also presented (Fig. 5).

**Table 3:** Prevalence and concentration ranges of antimicrobial residues present in chicken breast, thigh, and liver samples analyzed by HPLC compared with their maximum residue limits (MRLs).

Antimicrobial agents	Sample source	Number of tested samples	Number and (%) of positive samples	Mean ± SE^#^ (µg/kg)	Minimum level (µg/kg)	Maximum level (µg/kg)	MRL^*^ (µg/kg)	Number and (%) of positive samples over MRL	Number and (%) of positive samples below MRL
Enrofloxacin	Breast	60	30 (50%)	90.4 ± 24	10	247	100	9 (30%)	21 (70%)
	Thigh	60	33 (55%)	161.5 ± 44	8	554	100	21 (63.64%)	12 (36.36%)
	Liver	60	30 (50%)	1707.3 ± 686	39	6386	200	21 (70%)	9 (30%)
	Overall	180	93 (51.67%)	637.2 ± 253	8	6386		51 (54.84%)	42 (45.16%)
Oxytetracycline	Breast	60	36 (60%)	317.7 ± 198	38	2490	200	9 (25%)	27 (75%)
	Thigh	60	45 (75%)	964.1 ± 371	28	4320	200	18 (40%)	27 (60%)
	Liver	60	48 (80%)	10,625 ± 2660	320	41,460	600	45 (93.75%)	3 (6.25%)
	Overall	180	129 (71.67%)	4378.5 ± 1229	28	41,460		72 (55.81%)	57 (44.19%)
Sulfadimidine	Breast	60	9 (15%)	573.3 ± 226	425	950	100	9 (100%)	Nil
	Thigh	60	9 (15%)	47 ± 18	41	80	100	Nil	9 (100%)
	Liver	60	12 (20%)	3848.1 ± 886	1280	5200	100	12 (100%)	Nil
	Overall	180	30 (16.67%)	1489.4 ± 577	41	5200		21 (70%)	9 (30%)

^#^Mean ± SE of positive samples.

*MRL = Maximum Residue Limit. The MRL for enrofloxacin is according to the European Union (2010), while the MRLs for oxytetracycline and sulfadimidine are according to the Codex Alimentarius Commission (2024).

In the present study, enrofloxacin residues (fluoroquinolone group) were detected in 50% (30/60), 55% (33/60), and 50% (30/60) of chicken breast, thigh, and liver samples, respectively, with an overall prevalence of 51.7% (93/180) (Table 3). Likewise, Aslam et al. in Pakistan revealed that enrofloxacin residues were detected in 52% (39/75) of chicken meat samples, although the corresponding incidence in liver samples was 78.7% (59/75)^35^. Lower enrofloxacin incidence of 21% (33/160), 24% (39/160), and 36% (57/160) were detected in chicken breast, thigh, and liver samples tested in Bangladesh with the use of thin layer chromatography (TLC), respectively^36^. Enrofloxacin residues were also detected in chicken breast and pooled liver samples examined in Brazil, with low incidences of 23% (14/60) and 17% (1/6), respectively^37^. Similarly, 32.8% (20/61) of chicken muscle samples analyzed in Portugal were positive for enrofloxacin residues^38^. Enrofloxacin was detected at lower incidence rates of 12.5% (10/80) and 12.1% (7/58) in chicken meat samples from Lebanon using HPLC^39^ and Korea^40^ using LC-MS/MS, respectively. Fei et al. reported that only 1.87% of chicken meat samples analyzed with UPLC-MS/MS in China were positive for enrofloxacin, with the highest measured concentration reaching 2017 µg/kg^41^.

On the contrary, a much higher incidence of enrofloxacin was observed in Iran, where all analyzed chicken muscle and liver samples (100%, 180/180) were positive, with 8.9% (8/90) and 13.3% (12/90) of muscle and liver samples, respectively, exceeding the MRL set for enrofloxacin^42^. In Malaysia, 91.9% (34/37) of chicken muscle samples were positive for enrofloxacin, with concentrations ranging from 3.51 to 1734.61 µg/kg^43^. Another study in Egypt found enrofloxacin residues in 60% (6/10) of fresh domestic broiler muscle samples and 60% (6/10) of pooled giblet samples, including liver^44^.

**Fig. 4:**
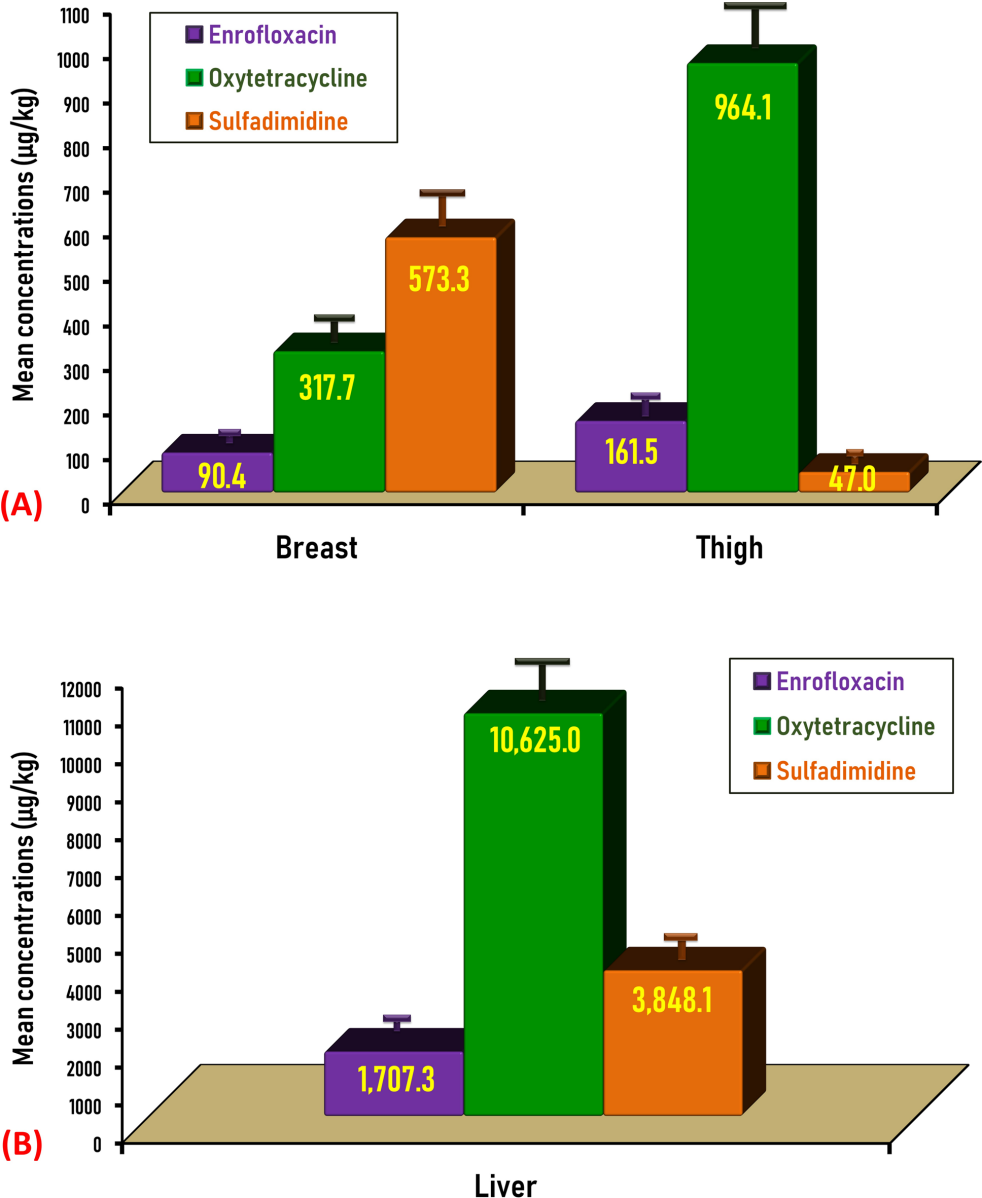
Mean concentrations of enrofloxacin, oxytetracycline, and sulfadimidine (µg/kg) in chicken breast and thigh samples (**A**). Mean concentrations of enrofloxacin, oxytetracycline, and sulfadimidine (µg/kg) in chicken liver samples (**B**).

In this study, enrofloxacin concentrations in chicken breast, thigh, and liver ranged from 10 to 247, 8 to 554, and 39 to 6386 µg/kg, respectively (Table 3). The corresponding mean ± SE values were 90.4 ± 8.2 µg/kg for breast, 161.5 ± 14.8 µg/kg for thigh, and 1707.3 ± 121.3 µg/kg for liver (Fig. 4). Enrofloxacin residues were significantly higher (*P* < 0.05) in liver tissue compared with breast and thigh, while no significant difference (*P* > 0.05) was observed between breast and thigh samples. Overall, enrofloxacin accumulated in chicken tissues in the following order: Liver > Thigh > Breast. A similar order was reported in Bangladesh^36^.

**Fig. 5:**
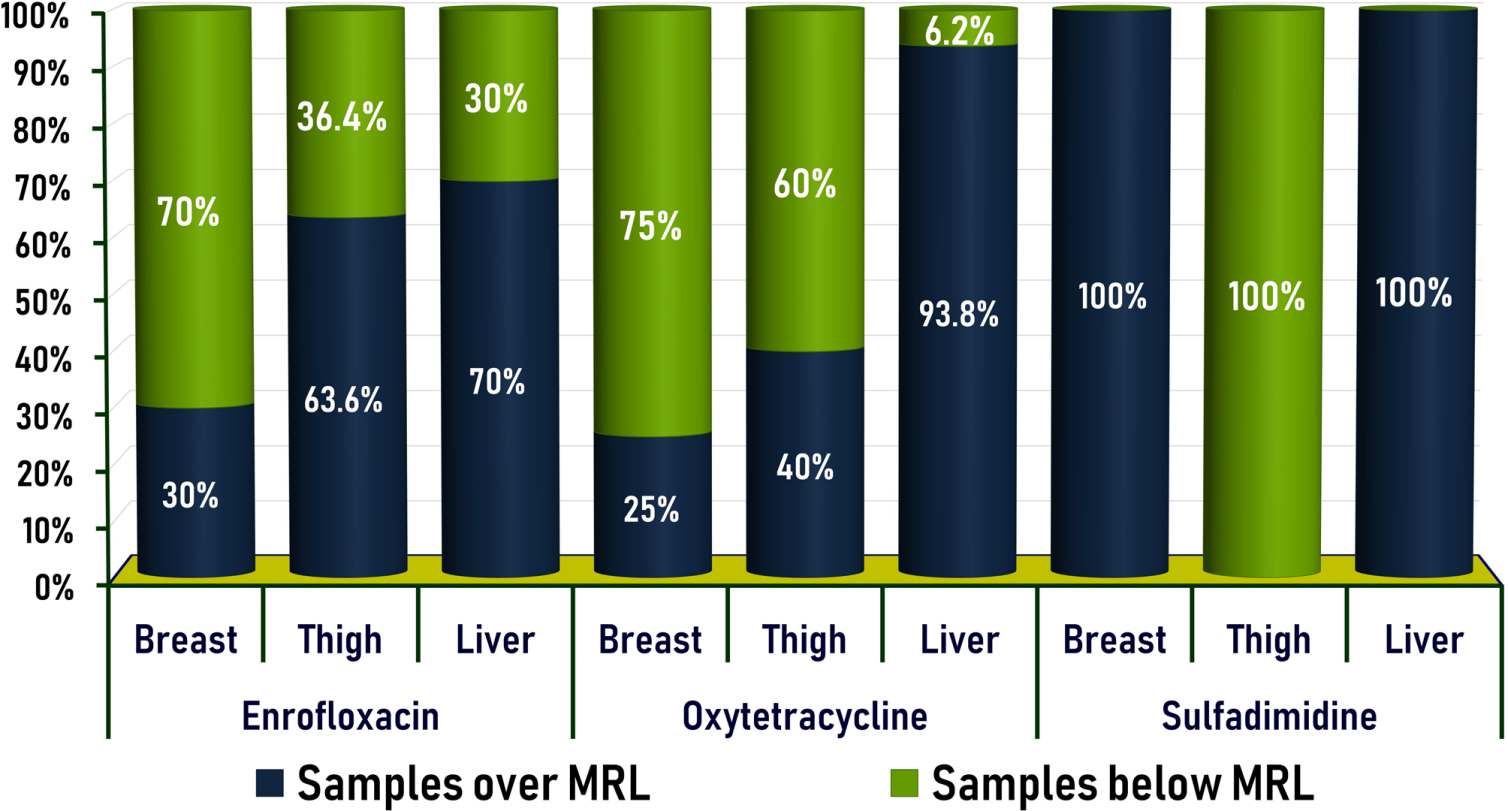
Comparison between chicken samples exceeding the MRLs versus the positive-compliant samples below MRLs.

Many other studies have reported higher concentration values. For instance, in Pakistan, the mean concentration of enrofloxacin was 208 µg/kg in broiler meat samples^35^, while in China, the highest concentration of enrofloxacin was 2017 µg/kg^41^. In Egypt, Khalafalla et al. reported substantially higher ENR residue levels, reaching 24,400 µg/kg in chicken carcasses and 38,850 µg/kg in the examined giblets analyzed by HPLC^44^. In contrast, a lower concentration of enrofloxacin was observed in other regions. For example, in Brazil, enrofloxacin concentrations ranged from 0.96 to 35.8 µg/kg in chicken breast and were 45.4 µg/kg in liver samples^37^. In Turkey, ENR levels in chicken breast meat ranged from 124.3 to 144.1 µg/kg, while drumstick samples contained 111.9 to 115.5 µg/kg^45^. Moreover, lower concentrations ranging from 0.35 to 170.5 µg/kg of enrofloxacin were reported in chicken meat in India^33^, Korea^40^, and Portugal^38^.

It was reported that 30% (9/30), 63.64% (21/33), and 70% (21/30) of enrofloxacin-positive chicken breast, thigh, and liver samples, respectively, exceeded the maximum residue limit (MRL) for enrofloxacin, as reported by Codex Alimentarius^25^ (Table 3; Fig. 5). Similarly, Aslam et al. reported that enrofloxacin residues exceeded the maximum residue limit (MRL) in 58.3% (21/36) of chicken meat samples and 71.2% (42/59) of chicken liver samples analyzed in Pakistan^35^. Nonetheless, lower incidence rates of 8.9% (8/90) and 13.3% (12/90) were observed in chicken muscle and liver samples tested in Iran, respectively, with enrofloxacin concentrations above MRL^42^. On the contrary, all ENR-positive chicken samples (100%, 6/6) contained residue levels exceeding the MRL of 100 µg/kg^45^.

### Prevalence and concentration of oxytetracycline residues (µg/kg) in chicken breast, thigh, and liver samples

HPLC analysis indicated that oxytetracycline was detected in 60% (36 out of 60), 75% (45 out of 60), and 80% (48 out of 60) of the chicken breast, thigh, and liver samples examined, respectively, with an overall prevalence of 71.7% (129/180) (Table 3). Similarly, 60% (6/10) of both chicken meat and giblet samples analyzed in Egypt contained oxytetracycline residues^44^. Additionally, with the use of UV-visible spectrophotometry, Bakr et al. reported that 69% (71/103) of chicken breast samples examined in Egypt had detectable OTC residues^46^.

Other studies have reported lower prevalence rates of oxytetracycline (OTC) residues. For instance, Salama et al. in Egypt detected OTC residues in 22% (11/50) of chicken breast samples, 18% (9/50) of thigh samples, and 24% (12/50) of liver samples analyzed^47^. Also in Egypt, Kamouh et al. detected OTC residues in 16% (4/25), 20% (5/25), and 32% (8/25) of chicken breast, thigh, and liver samples, respectively^48^. Likewise, lower OTC residues in chicken meat samples were determined in India^33^, Lebanon^39^, and China^41^ with incidences of 13.1% (44/336), 10% (8/80), and 5.51% (253/4591), respectively. On the other hand, a higher incidence of OTC was reported in Iran, where all examined chicken meat and liver samples tested (100%, 90/90) were OTC-positive, with mean concentrations of 88.2 µg/kg and 576.7 µg/kg, respectively^42^.

Oxytetracycline levels in chicken breast, thigh, and liver samples in this study ranged from 38 to 2490, 28 to 4320, and 320 to 41,460 µg/kg (Table 3) with mean ± SE values of 317.7 ± 22.4, 964.1 ± 111.5, and 10,625 ± 896.2 µg/kg, respectively (Fig. 4). Statistical analysis using ANOVA revealed that oxytetracycline residues were markedly greater (*P* < 0.05) in liver samples than in both breast and thigh tissues. In contrast, residue levels in breast and thigh did not differ significantly (*P* > 0.05). Overall, the distribution pattern of oxytetracycline in chicken tissues showed the highest accumulation in the liver, followed by the thigh, and the lowest levels in the breast (Fig. 4). A similar pattern was determined in Egypt by Kamouh et al.^48^.

Compared with the present findings, Salama et al. reported lower residual oxytetracycline levels, with concentrations ranging from 124 to 1914 µg/kg in chicken breast, 107 to 1897.5 µg/kg in thigh, and 103 to 2930 µg/kg in liver samples^47^. Similarly, Kamouh et al. recorded oxytetracycline concentrations ranging from 25.1 to 239.5 µg/kg in breast tissue, 31.6 to 326.2 µg/kg in thigh tissue, and 502.9 to 813.8 µg/kg in liver tissues^48^. Several other studies had also indicated comparatively lower OTC levels in chicken tissues. Haque et al. revealed that raw broiler chicken available in a local area of Bangladesh contained OTC with a concentration of 40–65 µg/kg in muscles and 69.4–109.5 µg/kg in liver^34^. Likewise, Chandrakar et al. reported a range of 50.9 to 211.4 µg/kg for OTC concentrations in chicken meat samples in India^33^, while Jammoul and El Darra detected OTC concentrations between 9.6 and 46.2 µg/kg with a mean concentration value of 22.6 µg/kg in chicken samples tested in Lebanon^39^. Additionally, in China, the highest OTC concentration identified in chicken meat samples was 410 µg/kg with a mean value of 1.78 µg/kg^41^, while in Iran, the OTC residual values ranged from 6.6 to 255.3 and 210 to 1362 in chicken meat and liver samples, respectively^42^. Conversely, Khalafalla et al. detected higher values for OTC of 16,680 µg/kg in chicken muscle samples from Egypt, although their liver samples exhibited lower OTC concentrations (2680 µg/kg)^44^.

It has been reported that 25% (9/36), 40% (18/45), and 93.75% (45/48) of oxytetracycline-positive chicken breast, thigh, and liver samples, respectively, exceeded the maximum residue limits (MRLs) estimated for oxytetracycline by Codex Alimentarius^25^(Table 3; Fig. 5). Similarly, 27.8% (25/90) of chicken meat samples and 95.6% (85/90) of liver samples tested in Iran showed OTC residues above MRLs^42^. Moreover, Kamouh et al. reported a nearly similar prevalence of oxytetracycline-positive samples exceeding the MRLs in chicken thigh samples (40%, 2/5) from Egypt, with a higher prevalence observed in breast samples from the same carcasses (50%, 2/4), whereas liver samples showed a lower incidence (75%, 6/8)^48^. On the contrary, Verma et al. reported that oxytetracycline levels exceeded the maximum permissible limits in 35.7% (5/14) of OTC-positive chicken meat samples and 31.6% (6/19) of OTC-positive liver samples in India^49^. Furthermore, Ahmed and Gareib^50^ reported a higher percentage of non-compliant samples in Egypt, with 70% (14/20) of oxytetracycline-positive chicken breast samples exceeding the permissible limits. In contrast, only 5.6% (4/71) of chicken breast samples in Egypt exceeded the MRLs^46^.

### Prevalence and concentration of sulfadimidine residues (µg/kg) in chicken breast, thigh, and liver samples

HPLC analysis revealed the presence of sulfadimidine (SDI) in 9 (15%), 9 (15%), and 12 (20%) of chicken breast, thigh, and liver samples tested in the present study, respectively, with a total incidence of 16.7% (30/180) (Table 3). Likewise, Lee et al. detected sulfonamide (sulfamethoxazole) residues in 6 of 58 (12.1%) chicken meat samples in Korea^40^. A similar incidence of 14% (4/25) was also detected for SDI by Abd El Razik et al. in the thigh samples collected from various chicken slaughterhouses in Egypt, although they determined a lower prevalence of 8% (2/25) of SDI residues in chicken breast, and a higher prevalence of 28% (7/25) in liver samples^51^. In this context, in Egypt revealed that 60% (6/10) of chicken meat and 10% (1/10) of pooled giblets, including liver, were positive for SDI^44^. In Nepal, however, sulfonamide residues were detected in 26.19% (11/42) and 7.14% (3/42) of chicken muscle and liver samples^52^. A much lower incidence of 3.75% (3/80) was detected for SDI residues in broiler chicken meat samples tested in Lebanon^39^.

In the present study, sulfadimidine residues in chicken tissues varied from 425 to 950 µg/kg in breast, 41 to 80 µg/kg in thigh, and 1280 to 5200 µg/kg in liver samples (Table 3), with mean ± SE concentrations of 573.3 ± 46.8 µg/kg for breast, 47 ± 5.2 µg/kg for thigh, and 3848.1 ± 365.5 µg/kg for liver (Fig. 4). Sulfadimidine residue levels were significantly higher (*P* < 0.01) in liver tissue compared to both breast and thigh, along with a significant difference (*P* < 0.01) between breast and thigh samples. Sulfadimidine residues in chicken tissues exhibited the following distribution pattern: liver > breast muscle > thigh muscle. The liver concentration was 81.9-fold higher than that detected in the thigh and 6.7-fold higher than that in the breast. Notably, the concentration in the breast muscle was 12.2-fold greater than that in the thigh, since the breast is the site of sulfadimidine injection. On the contrary, Abd El Razik et al. reported a different distribution pattern in Egypt, with SDI residues occurring in the order Liver > Thigh > Breast^51^.

Previous studies in Egypt reported different SDI concentrations in chicken tissues. Mean ± SE concentrations of 47.9 ± 1.7 µg/kg, 69.4 ± 2.6 µg/kg, and 149.5 ± 7.9 µg/kg were determined for sulfadimidine in chicken breast, thigh, and liver samples, respectively^51^. Moreover, Khalafalla et al. detected a high sulfadimidine concentration of 7170 and 160 µg/kg in chicken carcasses and pooled giblets, including liver, respectively^44^. Much lower mean SDI concentration of 0.4 µg/kg was determined in broiler chicken meat samples tested in Lebanon^39^, and also lower mean SDI concentrations of 16.12 and 15.87 µg/kg were reported in chicken meat and liver samples tested in Nepal, respectively^52^. Likewise, Kamel et al. reported a lower mean ± SE sulfadimidine concentration of 75.54 ± 15.64 µg/kg in Egyptian broiler chicken meat samples^53^.

According to the current study, all sulfadimidine-positive chicken breast (100%, 9/9) and liver samples (100%, 12/12), contained residue levels exceeded the MRL established by the European Union^54^, on the other hand, none of the thigh samples exceeded the MRLs (Table 3; Fig. 5). In contrast, Abd El Razik et al. revealed that 50% (2/4) of SDI-positive thigh samples and 71.4% (5/7) of SDI-positive liver samples exceeded the MRLs, while none of the SDI-positive chicken breast samples exceeded such limit^51^. Furthermore, Mehtabuddin et al. reported that sulfonamide residues exceeded the MRL in 53.8% (7/13) of positive chicken breast samples analyzed in Pakistan^55^.

### Coexistence of antibiotic residues in chicken meat and liver

Of the 60 chicken carcasses examined, 24 (40%) contained enrofloxacin residues in their breast, thigh, and liver. Additionally, 3 (5%) chicken carcasses contained ENR residues in both breast and liver tissues, 9 (15%) carcasses contained such an antibiotic in the thigh meat only, and 3 (5%) carcasses had it only in the liver. Conversely, 21 (35%) carcasses were negative for enrofloxacin residues.

Oxytetracycline residues were identified in each of the breast, thigh, and liver tissues of 24 (40%) chicken carcasses out of the 60 carcasses tested. Moreover, OTC residues were detected in both breast and thigh tissues of 3 (5%) carcasses, as well as in both breast and liver tissues of 9 (15%) carcasses, and in both thigh and liver tissues of 9 (15%) carcasses. Moreover, 6 (10%) chicken carcasses had OTC only in their thigh tissue, and another 6 (10%) carcasses had OTC only in their liver tissue. On the other hand, only 3 (5%) carcasses were negative for oxytetracycline residues.

Sulfadimidine residues were present in breast, thigh, and liver tissues of 9 (15%) chicken carcasses, and only in the liver of 3 (5%) carcasses. In contrast, the majority (48; 80%) of chicken carcasses were negative for sulfadimidine residues.

Residues of all three antibiotics analyzed could be detected in 5% of breast, thigh, and liver samples tested, while 50%, 50%, and 40% of thigh, liver, and breast samples contained residues of two antibiotics, respectively. On the other hand, only 25%, 15%, and 10% of breast, thigh, and liver samples, respectively, were negative for the three antibiotics tested (Table 4), indicating a higher overall burden of antibiotic residues in these tissues. The present findings were inconsistent with Chandrakar et al., who reported that 9.5% of chicken meat samples analyzed in India were positive for two or more antibiotics^33^. Similarly, in China, 4.8% of the chicken meat samples tested contained at least two antibiotics^41^.

**Table 4:** Coexistence of antibiotic residues in breast, thigh, and liver samples of the chicken carcasses tested (*n* = 60).

Tissue type	Coexistence of 3 antibiotics Number and (%)	Coexistence of 2 antibiotics Number and (%)	Samples positive for only 1 antibiotic Number and (%)	Antibiotic negative samples Number and (%)
Breast (*n* = 60)	3 (5%)	24 (40%)	18 (30%)	15 (25%)
Thigh (*n* = 60)	3 (5%)	30 (50%)	18 (30%)	9 (15%)
Liver (*n* = 60)	3 (5%)	30 (50%)	21 (35%)	6 (10%)
Total (*n* = 180)	9 (5%)	84 (46.67%)	57 (31.67%)	30 (16.67%)

The high prevalence of antimicrobial residues detected in this study may be attributed to the extensive use of antibiotics in poultry production, inappropriate dosing practices, and failure to observe recommended withdrawal periods before slaughter. The higher residue concentrations observed in liver samples are likely due to the liver’s central role in drug metabolism and accumulation.

### Estimation of health risk and hazard quotient (HQ) associated with enrofloxacin, oxytetracycline, and sulfadimidine residues in chicken

The presence of antibiotic residues in food products is regarded as a public health concern, as some residues have been linked to adverse health effects, including allergic reactions, mutations, toxic effects, disruption of the intestinal microbiota, and the development of antimicrobial resistance^18^. The Centre for Disease Control and Prevention (CDC) and the U.S. Food and Drug Administration (FDA) have identified antibiotic-resistant pathogens as one of the most serious global health threats of the last few decades. The main contributor to the development of such resistance is the improper or abusive use of antibiotics in both human and veterinary medicine. Other health effects in consumers, such as gastrointestinal and neurological disorders, are less consistently reported^56,57^.

Estimated Daily Intake (EDI) is defined as the daily amount of antibiotic residues ingested by an individual from consuming chicken meat, adjusted for body weight. Chandrakar et al. calculated the EDI for different age groups by multiplying the mean concentration of antibiotic residues in chicken meat (MC, µg/kg) by the daily consumption of chicken meat (DC) and then dividing the result by the individual’s body weight (BW, kg)^33^. The results are expressed in µg of antibiotic per kg of body weight per day. The use of EDI in HQ estimation provides a more realistic assessment of exposure by relating actual intake levels to the established safety threshold. An HQ value equal to or greater than 1 indicates a potential risk of adverse health effects, whereas an HQ value below 1 suggests negligible or insignificant risk^58^.

In Egypt, the per capita daily intake of chicken is approximately 65.2 g/day (0.0652 kg/day) https://worldpopulationreview.com/country-rankings/chicken-consumption-by-country. Since the annual consumption of chicken in Egypt is equivalent to 19 chicken carcasses weighing about 1.25 kg, with an average weight of chicken breast of approximately 450 g and thigh of 400 g, the corresponding daily consumption is estimated to be 23.4 g/day (0.0234 kg/day) for chicken breast and 20.8 g/day (0.0208 kg/day) for chicken thigh. The chicken liver is predicted to weigh about 30 g; hence, the daily chicken liver consumption is estimated at 1.6 g/day (0.0016 kg/day). Moreover, the EDI is calculated assuming body weights of 34 kg, 77.1 kg, and 79.1 kg for school-aged children (5–14 years), women (15–59 years), and men (15–59 years), respectively (Table 5). The Acceptable Daily Intake (ADI) represents the maximum amount of antimicrobial residue that can be consumed each day over a lifetime without posing considerable health risks, expressed per unit of body weight. The acceptable daily intake (ADI) value for enrofloxacin is 0–2 µg/kg bw as stated by JECFA^59^, while the ADI for oxytetracycline and sulfadimidine are 0–30 µg/kg bw and 0–50 µg/kg bw, respectively, according to the Codex Alimentarius Commission^25^.

**Table 5:** Health risk estimation of enrofloxacin, oxytetracycline, and sulfadimidine residues in chicken breast, thigh, and liver.

Antibiotics	Sample source	^1^EDI (µg/kg bw*) for the different age groups of the Egyptian consumers			^2^ADI (µg/kg bw)	^3^HQ (Hazard Quotient) for the different age groups of the Egyptian population		
		Schoolchildren (5–14 years; 34 kg)	Women (15–59 years; 77.1 kg)	Men (15–59 years; 79.1 kg)		Schoolchildren (5–14 years; 34 kg)	Women (15–59 years; 77.1 kg)	Men (15–59 years; 79.1 kg)
Enrofloxacin	Chicken breast	0.062	0.027	0.027	2^a^	0.031	0.014	0.014
	Chicken thigh	0.099	0.044	0.042		0.050	0.022	0.021
	Chicken liver	0.080	0.035	0.035		0.040	0.018	0.018
Oxytetracycline	Chicken breast	0.219	0.096	0.094	30^b^	0.0073	0.0032	0.0031
	Chicken thigh	0.590	0.260	0.254		0.0197	0.0087	0.0085
	Chicken liver	0.500	0.220	0.215		0.0167	0.0073	0.0072
Sulfadimidine	Chicken breast	0.395	0.174	0.170	50^b^	0.0079	0.0035	0.0034
	Chicken thigh	0.029	0.013	0.012		0.0006	0.0003	0.0002
	Chicken liver	0.181	0.080	0.078		0.0036	0.0016	0.0016

^1^EDI (Estimated daily intake) = MC × DC /BW. MC is the mean concentration of antimicrobial residues (µg/ kg). The daily intake of chicken for Egyptians is approximately 23.8 kg/capita/year-Egypt https://worldpopulationreview.com/country-rankings/chicken-consumption-by-country EDI is calculated by the equation set by Chandrakar et al. (2023). EDI is expressed as µg/kg BW.

*BW is the average body weight for the different age groups of the Egyptian population, which is estimated at 34 kg for schoolchildren aged 5–14 years according to Egyptian growth charts (https://ia804602.us.archive.org/2/items/egyptian-growth-charts/Egyptian%20growth%20charts.pdf*)*, and 77.1 kg for women and 79.1 kg for men aged 15–59 years according to the Ministry of Health and Population, Cairo, Egypt (https://dhsprogram.com/pubs/pdf/FR313/FR313.pdf*).*

^2^ADI= Acceptable Daily Intake; ^a^ JECFA, (1997); ^b^ Codex Alimentarius Commission (2024).

^3^HQ (Hazard Quotient): HQ = EDI / ADI. An HQ of < 1 indicates acceptable, low risk, while > 1 suggests a considerable risk. HQ is calculated by the equation determined by JECFA (2002) and Chandrakar et al. (2023).

The highest average daily intake of antibiotics from chicken was recorded among schoolchildren with levels of 0.029–0.590 µg/kg bw/day, followed by women with intake levels between 0.013 and 0.260 µg/kg body weight/day, while the lowest EDI values were observed in men, ranging from 0.012 to 0.254 µg/kg body weight/day (Table 5). In the current study, the EDI for the antibiotics tested was determined across different age groups of the Egyptian population. The EDI value of oxytetracycline was the highest, while that of sulfadimidine was the lowest. This can be attributed to the high concentrations of OTC detected in the present study, which likely contributed to the high EDI values across all age groups. The present results were consistent with findings reported in India, where children were identified as the primary risk group for exposure to enrofloxacin, oxytetracycline, and sulfonamides through consumption of chicken tissues^33^. Likewise, a Chinese study identified children as the most vulnerable population group to antibiotic exposure due to their lower body weights^41^.

Hazard Quotient (HQ) is a risk assessment method used to evaluate the potential health risk from exposure to a chemical substance. The Hazard Quotient can be calculated based on the EDI and ADI^33,59^. The HQ values of enrofloxacin, oxytetracycline, and sulfadimidine ranged from 0.0006 to 0.050 for schoolchildren, from 0.0003 to 0.022 for women, and from 0.0002 to 0.021 for men, respectively. Chicken thigh exhibited the highest QH for enrofloxacin residues and the lowest QH for sulfadimidine for all age groups (Table 5). Higher HQ values were detected for the schoolchildren group, and ENR showed the highest HQ among the antibiotics, followed by OTC and SDI. The HQ values for different age groups were below 1.0, indicating no serious adverse health effects from individual exposure to these antibiotics via chicken meat and liver consumption. Similarly, lower HQ values than the toxicological reference values for enrofloxacin and oxytetracycline in chicken tissue were detected in India^33^ and in China^41^. Although the HQ values were negligible for both children and adults, the values observed in children were relatively higher, and adults exhibited greater safety margins than children. Therefore, precise and continued monitoring of antibiotic use in the poultry industry is important to protect vulnerable populations, prevent possible adverse effects, and limit the development of antibiotic resistance.

## Conclusion

The majority of chicken carcasses tested were positive for enrofloxacin, oxytetracycline, and sulfadimidine, with overall higher concentrations in liver samples than in chicken breast and thigh samples. More than half of the samples containing antibiotic residues exceeded the MRLs, indicating a serious risk to public health. Although the Hazard Quotient was less than 1, the accumulation of such antimicrobial residues through poultry consumption may pose a potential risk to human health. Therefore, regulatory authorities need to monitor and regulate the use of veterinary drugs in the poultry industry, with consideration of withdrawal periods, to minimize antibiotic residues in food of animal origin, and to control the spread of antibiotic-resistant bacteria, thereby protecting public health.

## Data Availability

All data generated or analyzed during this study are included in this published article.
